# RING finger ubiquitin E3 ligase gene *TaSDIR1-4A* contributes to determination of grain size in common wheat

**DOI:** 10.1093/jxb/eraa271

**Published:** 2020-06-01

**Authors:** Jingyi Wang, Ruitong Wang, Xinguo Mao, Jialing Zhang, Yanna Liu, Qi Xie, Xiaoyuan Yang, Xiaoping Chang, Chaonan Li, Xueyong Zhang, Ruilian Jing

**Affiliations:** 1 National Key Facility for Crop Gene Resources and Genetic Improvement/Institute of Crop Science, Chinese Academy of Agricultural Sciences, Beijing, China; 2 Institute of Genetics and Developmental Biology, Chinese Academy of Sciences, Beijing, China; 3 Shanghai Jiao Tong University, China

**Keywords:** Association analysis, functional marker, RING E3 ubiquitin ligase, TGW, *Triticum aestivum*, VIGS, wheat

## Abstract

Salt and drought-induced RING finger1 (SDIR1) is a RING-type E3 ubiquitin ligase that plays a key role in ABA-mediated responses to salinity and drought stress via the ubiquitination pathway in some plant species. However, its function in wheat (*Triticum aestivum*) is unknown. Here, we isolated a *SDIR1* member in wheat, *TaSDIR1-4A*, and characterized its E3 ubiquitin ligase activity. DNA polymorphism assays showed the presence of two nucleotide variation sites in the promoter region of *TaSDIR1-4A*, leading to the detection of the haplotypes *Hap-4A-1* and *Hap-4A-2* in wheat populations. Association analysis showed that *TaSDIR1-4A* haplotypes were associated with 1000-grain weight (TGW) across a variety of different environments, including well-watered and heat-stress conditions. Genotypes with *Hap-4A-2* had higher TGW than those with *Hap-4A-1*. Phenotypes in both gene-silenced wheat and transgenic Arabidopsis showed that TaSDIR1-4A was a negative regulator of grain size. Gene expression assays indicated that *TaSDIR1-4A* was most highly expressed in flag leaves, and expression was higher in *Hap-4A-1* accessions than in *Hap-4A-2* accessions. The difference might be attributable to the fact that TaERF3 (ethylene response factor) can act as a transcriptional repressor of *TaSDIR1-4A* in *Hap-4A-2* but not in *Hap-4A-1*. Examination of modern wheat varieties shows that the favorable haplotype has been positively selected in breeding programs in China. The functional marker for *TaSDIR1-4A* developed in this study should be helpful for future wheat breeding.

## Teaser

The functional E3 ligase TaSDIR1-4A is a negative regulator of grain size in wheat, and a molecular marker is developed that distinguishes the more favorable of the two halotypes that exist.

## Introduction

Given rapid increases in population together with climate change, we could soon witness global food shortages (Boyer, 1982; Godfray *et al.*, 2010; Tilman *et al.*, 2011). Grains are the most important food sources for humans (Zuo and Li, 2014; Li *et al.*, 2018), and grain size is an important component of grain weight. Therefore, an understanding of the mechanisms underlying seed development is important for improvement of grain yield in crop species. Recent studies have shown that ubiquitin-proteasome pathways are involved in control of seed size (Li and Li, 2016; Nadolska-Orczyk *et al.*, 2017; Kelley, 2018; Li *et al.*, 2019*b*; Xu and Xue, 2019), and one of these, the DA1 pathway, plays a key role in controlling seed size in Arabidopsis by regulating cell proliferation in the integuments (Disch *et al.*, 2006; Xia *et al.*, 2013*a*; Du *et al.*, 2014; Li and Li, 2016; Dong *et al.*, 2017). This pathway of control of seed size is highly conserved among species (Su *et al.*, 2011; Wang *et al.*, 2017; Xie *et al.*, 2018). For example, *Grain Width and Weight2* (*GW2*), a major quantitative trait locus (QTL) in rice, is a homolog of *DA2*, a RING-type E3 ubiquitin ligase gene (Song *et al.*, 2007; Xia *et al.*, 2013*a*). *GW2* homologs in maize and wheat are also involved in the control of grain size (Li *et al.*, 2010*b*; Bednarek *et al.*, 2012; Zhang *et al.*, 2018).

Another RING-type E3 ubiquitin ligase, salt and drought-induced RING finger1 (SDIR1), has been shown to primarily participate in stress responses in numerous species, including Arabidopsis (Zhang *et al.*, 2007, 2015), maize (Xia *et al.*, 2012; Liu *et al.*, 2013), rice (Gao *et al.*, 2011), tobacco (Xia *et al.*, 2013*b*), and grape (Tak and Mhatre, 2013), while others have been identified to not only be involved in stress responses, but also in plant growth and grain development (Sun *et al.*, 2019). In wheat, there are two AtSDIR1 homologs, TaSDIR1-4A and TaSDIR1-4D, and the latter might be involved in abiotic stress responses (Wang *et al.*, 2018). To date, the role of *TaSDIR1* in grain size development in wheat is unknown.

Based on gene-sequence polymorphisms, candidate gene association analysis has been shown to be an efficient approach to reveal relationships between gene/polymorphic loci and traits, and it has been widely used in many crop species, including maize (Wang *et al.*, 2016*b*) and wheat (Zhang *et al.*, 2017). A series of elite alleles and molecular markers associated with important traits have been discovered using this method (Cao *et al.*, 2020), and pyramiding these elite alleles through marker-assisted selection (MAS) will greatly accelerate the process of wheat breeding. Hence, finding elite alleles associated with important agronomic traits is considered as being fundamental to wheat genetic improvement.

In the present research, we identified the RING-type E3 ubiquitin ligase gene *TaSDIR1-4A* and performed association analysis using multiple wheat populations, which demonstrated that it was significantly associated with 1000-grain weight. The favorable haplotype/allele has been positively selected for in Chinese wheat breeding programs, and the functional marker that we have developed for this haplotype will be beneficial in future programs.

## Materials and methods

### Plant materials

The common wheat (*Triticum aestivum*) cultivar Hanxuan 10 (H10) was used for cloning and structural analysis of the *TaSDIR1* genes. A diverse wheat population of 32 accessions previously screened with 209 SSR markers (Zhang *et al.*, 2013) was used for gene sequencing and detection of nucleotide polymorphisms (Supplementary Table S1 at *JXB* online).

Four wheat populations were used in this study, as follows. Population 1 was a doubled-haploid (DH) population with 150 lines developed from the cross H10 × Lumai 14 (L14) and was used for genetic mapping. H10 was selected from more than 20 000 wheat accessions due to its strong drought tolerance, whilst L14 is a high-yielding cultivar adapted to well-watered and fertile conditions, and was widely grown in northern China during the 1990s. Population 2 (262 accessions) was used for association analysis and consisted of 209 modern varieties, 43 advanced lines, and 10 landraces (Zhang *et al.*, 2013). Population 3 comprised 157 landraces, and Population 4 comprised 348 modern cultivars mainly from a Chinese wheat mini-core collection that represents more than 70% of the genetic diversity of the entire Chinese germplasm collection (Hao *et al.*, 2011). Populations 3 and 4 were also used for determination of haplotype frequencies in 10 different wheat production zones covering the whole of China (Zhang *et al.*, 2002).

### Growth conditions and measurement of agronomic traits

Population 1 was planted in 10 environments (year×site×water regime combinations) at Shunyi (40°230′N; 116°560′E) and Changping (40°130′N; 116°130′E) in Beijing during the 2015–2016 and 2016–2017 growing seasons. Population 2 was planted in 10 environments (year×site×water regime×heat stress combinations), including the same sites as Population 1, during 2010–2011, 2011–2012, and 2012–2013 for measurement of the following agronomic traits: plant height (PH), spike length (SL), peduncle length (PLE), length of penultimate node (LPN), number of spikes per plant (NSP), number of spikelets per spike (NSS), number of grains per spike (NGS), and 1000-grain weight (TGW). Two water regimes were applied at each site, namely well-watered (WW) and rain-fed (drought stress, DS). The WW plots were irrigated with 750 m^3^ ha^–1^ (75 mm) water at pre-overwintering, jointing, flowering, and grain-filling, whereas the DS plots were rain-fed only. The rainfalls were 131 mm in the 2010–2011 growing season, 180 mm in 2011–2012, 158 mm in 2012–2013, 161 mm in 2015–2016, and 173 mm in 2016–2017. A heat-stress (HS) treatment was applied 1 week post-anthesis at Shunyi by positioning a plastic film supported by steel frames over the plots in the field (Li *et al.*, 2019*a*). Populations 3 and 4 were planted in three environments, at Luoyang (34°610′N; 112°450′E) in Henan province in 2002 and 2005, and at Shunyi in 2010. In all cases, each accession was planted in a 2-m, 4-row plot with a row spacing of 30 cm and 40 seeds per row. Five plants in the middle of each plot were sampled for phenotyping at maturity.

### Isolation of *TaSDIR1-4A* and construction of a phylogenetic tree

The reference sequence of *TaSDIR1-4A* was obtained from the URGI website (https://wheat-urgi.versailles.inra.fr/). Genome-specific primers were designed based on the gene sequence (all primers are listed in Supplementary Table S2). Genomic DNA and cDNA from cv. H10 were used as templates. The gene structure of *TaSDIR1-4A* was determined using Lasergene 7.1.0 (DNASTAR, Inc., Madison, WI, USA) by alignment of the amplified cDNA and genomic DNA sequences. A neighbor-joining phylogenetic tree was constructed using the MEGA software v5.2 (https://www.megasoftware.net/).

### E3 ubiquitin ligase activity assays

The full-length *TaSDIR1-4A* ORF (840 bp) was cloned into a pMAL-C5X vector that has a maltose-binding protein (MBP) tag through seamless DNA cloning using In-Fusion^®^ HD Cloning Plus (638910, TaKaRa). A *TaSDIR1-4A* mutant (His-244 to Tyr) containing a mutation in the RING finger domain was constructed using a Site-directed Mutagenesis Kit (B639281, Sangon Biotech, Beijing) according to the manufacturer’s protocol. MBP, MBP-AtSDIR1, MBP-TaSDIR1-4A, and MBP-TaSDIR1-4A^H244Y^ were prepared and purified in *Escherichia coli*. MBP was used as a negative control, and MBP-AtSDIR1 was the positive control. *In vitro* E3 ligase assays were performed as described by Xie *et al.* (2002). Briefly, E1 (from wheat, 50 ng), E2 (UBCh5b, 100 ng), E3 (1μg), and 6×His tag ubiquitin (Ub, 4 μg) were mixed and incubated at 30 °C for 60 min. The mixture was separated by SDS-PAGE and blotted onto PVDF membranes (Millipore, IPVH00010). Anti-Ub and anti-MBP antibodies were used and bands were detected using a Thermo Pierce ECL (NCI4106) according to the manufacturer’s instructions.

### Sequence polymorphisms in *TaSDIR1-4A*

Nucleotide polymorphisms in *TaSDIR1-4A* sequences were detected by scanning a small diverse panel of 32 common wheat accessions. Genomic DNA was extracted from young leaves of 10-d-old seedlings using the CTAB method. *TaSDIR1-4A* fragments were amplified using primers specific to the A genome. Target bands were purified and cloned into the *pEASY*-Blunt vector, and transformed into *Trans*1-T1 phage-resistant chemically competent cells (CD501-03, Beijing Trans Gen Biotech Co., Ltd.) using the heat-shock method. Twelve positive clones were randomly selected for sequencing using a 3730 XI DNA Analyzer (ABI). *TaSDIR1-4A* nucleotide sequence polymorphisms were obtained using the SeqMan program in Lasergene 7.10 (DNASTAR).

### Functional marker development

A functional marker was developed based on a SNP (G/A) at position –395 bp, by mismatching bases in the dCAPS primer to create a restriction site. The first-round PCR product obtained by using primers specific to the A genome was used as a template for the second round of PCR, which was performed as follows: 95 °C for 5 min, followed by 33 cycles of 95 °C for 30 s, annealing 58 °C for 30 s, and extension at 72 °C for 30 s, with a final extension of 72 °C for 10 min. The PCR products were digested with restriction enzyme at 37 °C for 2 h and then separated by electrophoresis in 4% agarose gels.

### Chromosome location and linkage analysis

Diploid and tetraploid wheat relatives with various genomes and a set of nullisomic-tetrasomic lines of the common wheat cultivar Chinese Spring were used to identify the genomic origin and chromosome location of *TaSDIR1-4A*. The H10 × L14 DH population, and the IciMapping V4.1 software (www.isbreeding.net/software/) were used for gene mapping and linkage analysis.

### Population structure and association analysis

The population structure of Population 2 has previously been scrutinized with STRUCTURE v2.3.4 using data from 209 SSR markers evenly distributed on the 21 chromosomes in wheat (Li *et al.*, 2012*b*). The two subpopulations comprised 110 and 152 accessions. Association mapping was performed using the mixed linear model in TASSEL v2.1 (https://www.maizegenetics.net/tassel), which accounted for population structure. One-way ANOVA was performed in SPSS 19.0 to identify significant associations between gene haplotypes and agronomic traits. Tukey tests were used to determine significant differences at *P*=0.05.

### Virus-induced gene silencing (VIGS)

A series of recombinant barley stripe mosaic virus (BSMV) vectors was constructed. A non-conserved T*aSDIR1-4A* fragment of 253 bp spanning the 3´-terminal sequence and 3´-UTR region was selected to construct the BSMV vector BSMV:TaSDIR1-4A. This target region was compared with the Chinese Spring genome assembly v1.0 sequence (http://plants.ensembl.org/Triticum_aestivum/Tools/Blast) to ensure its specificity. A BSMV vector containing the GFP sequence was used as a control. To construct recombinant BSMV, the BSMV-γ vector was digested at the *Not* I restriction site, and the *TaSDIR1-4A* fragment amplified from the cDNA of Chinese Spring was inserted into the vector by seamless DNA cloning. The BSMV was inoculated onto leaves of jointing wheat plants following the procedures described in Wei *et al.* (2019). Flag leaves from BSMV-infected plants were collected at anthesis for determination of gene expression patterns. At maturity, seeds were collected, dried, and kernel size and weight were determined on samples of at least 200 seeds using an automatic seed measurement device (SC-A1, WSeen). Results were obtained from independent experiments.

### Production of transgenic Arabidopsis *TaSDIR1*-4A-overexpression lines

Seeds of the *sdir1-1* (SALK_052702) mutant were obtained from the Arabidopsis Biological Resource Center (https://abrc.osu.edu/. The full-length cDNA of *TaSDIR1-4A* with the 35S promoter was inserted into the modified pCAMBIA1300 vector at the *Xba* I and *Kpn* I restriction enzyme sites. *Agrobacterium* harboring *TaSDIR1-4A* and empty vectors were transferred into wild-type and *sdir1-1* Arabidopsis flowers using the floral dip method to obtain transgenic lines (Clough and Bent, 1998). The seed sizes of homozygous T_3_ generation plants were measured using a SC-A1 device.

### Expression of *TaSDIR1-4A* in wheat

The H10 cultivar was used for expression pattern analysis of *TaSDIR1-4A*. At the flowering stage, spikes, flag leaves, stems, nodes, root tissue at the stem base, and root tissues at different depths were sampled for spatial-temporal analysis of expression patterns (Wang *et al.*, 2019).

Twelve accessions of each of the two different haplotypes were randomly selected from each of Populations 1 and 2 (Supplementary Table S3). Flag leaves at anthesis were sampled from field-grown plants and used to assess the expression levels.

Based on pilot experiments, 2-week-old wheat seedlings were sprayed with 50 μM ABA solution or treated at 250 mM NaCl for 0.5–72 h. Whole plants were then sampled for detection of stress-induced expression of *TaSDIR1-4A*.

### Yeast one-hybrid assays

Yeast one-hybrid assays were performed to check the binding of the ethylene response factor DNA binding domain (TaERF-BD) to the *TaSDIR1-4A* promoter region. The pB42AD vector was digested with restriction enzyme *Not* I in order to insert the TaERF-BD. The *TaERF-BD* fragment was then cloned into the vector through seamless DNA cloning. Six fragments of the *TaSDIR1-4A* promoter region were cloned into pLacZi as the reporter gene plasmid as follows. For the H10 construct, a 588-bp (–757 to –170 bp) fragment from the *TaSDIR1-4A* promoter region of H10 (*Hap-4A-1*) was amplified and cloned into pLacZi by seamless DNA cloning. For the L14 construct, a 586-bp (–755 to –170 bp) fragment (*Hap-4A-2*) was amplified and cloned using the same method. For the ACC-H10 Box construct, the target fragment contained the triplicates of the ACC Box (32 bp, –418 to –387 bp from H10). For the GCC-H10 Box construct, the same fragment with an A-to-G mutation was used. For the GCC-L14 Box construct, the target fragment contained the triplicates of the GCC Box (32 bp, –418 to –387 bp from L14). For the ACC-L14 box construct, the same fragment with a G-to-A mutation was used. These four vectors were separately cloned into pLacZi by whole-gene synthesis at the Beijing Genomics Institute (BGI). Plasmids for the pB42AD vector with the TaERF-BD were co-transformed with the six reporter gene constructs into yeast strain EGY48 using standard yeast transformation methods (Lin *et al.*, 2007). The yeast was incubated on media lacking Ura and Leu with x-α-gal. Interactions between TaERF-BD and each promoter fragment were tested by LacZ staining (Wang *et al.*, 2016*a*).

### Purification of the TaERF-BD protein and electrophoretic mobility shift assays (EMSAs)

*TaERF-BD* cDNA (219 bp) was fused into the pGEX-4T1 vector, which has a glutathione *S*-transferase (GST) tag, using seamless DNA cloning at an *Eco*R I site using In-Fusion^®^ HD Cloning Plus. TaERF-BD protein expression in *Escherichia coli* BL21 cells was induced by 0.5 mM isopropyl-β-d-thiogalactoside (IPTG) at 28 °C for 6 h, and it was purified using glutathione–Sepharose 4B (52-2303-00, GE Healthcare). Unlabeled GCC-L14 and ACC-L14 Boxes, biotin-labelled GCC-L14 and ACC-L14, and GCC-H10 and ACC-H10 Boxes and their reverse complementary sequences were synthesized and annealed as probes. EMSA was carried out using a LightShift^®^ Chemiluminescent EMSA Kit (20148, Thermo Scientific) according to the manufacturer’s instructions.

### Dual-luciferase assays of transformed tobacco leaves

The full-length cDNAs of *TaERF3* and *TaERF115* were amplified using the primers TaERF3-1300-F/R and TaERF115-1300-F/R (Supplementary Table S2) and separately cloned into the effector vector pCAMBIA1300 under the control of CaMV 35S. The *TaSDIR1-4A* promoter fragment was amplified from H10 and L14 using the primers Hap-LUC-F/R (Supplementary Table S2) and separately ligated into the reporter vector pGreen II 0800-LUC. The effector and reporter constructs were transformed into GV3101 cells using pSoup, and transformed into leaves of 4-week-old *Nicotiana tabacum* plants by co-infiltration. The activities of firefly luciferase (LUC) and Renilla luciferase (REN) were measured using the Dual-Glo^®^ Luciferase Assay System (E2920, Promega) with a multimode reader (TriStar^2^ S LB942) at 48 h after infiltration. The promoter activity was calculated as the ratio of LUC to REN, and the ratio in leaves transformed with the empty vector (pCAMBIA1300/pGreen II 0800-LUC) was set to 1.

## Results

### TaSDIR1-4A is a functional E3 ligase

Two copies of *TaSDIR1* were isolated from the chromosome group 4 of subgenomes A and D in wheat, and hence were named as *TaSDIR1-4A* and *TaSDIR1-4D*. *TaSDIR1-4D* is known to be involved in responses to abiotic stress (Wang *et al.*, 2018). The cDNA sequence of *TaSDIR1-4A* obtained from cv. H10 consisted of eight exons and seven introns and encoded a protein containing 280 amino acids. TaSDIR1-4A, belonging to the RING finger domain family, contained two transmembrane domains in the N-terminal region, and a C3H2C3-type RING domain in the C-terminal region (Supplementary Fig. S1).

**Fig. 1.**
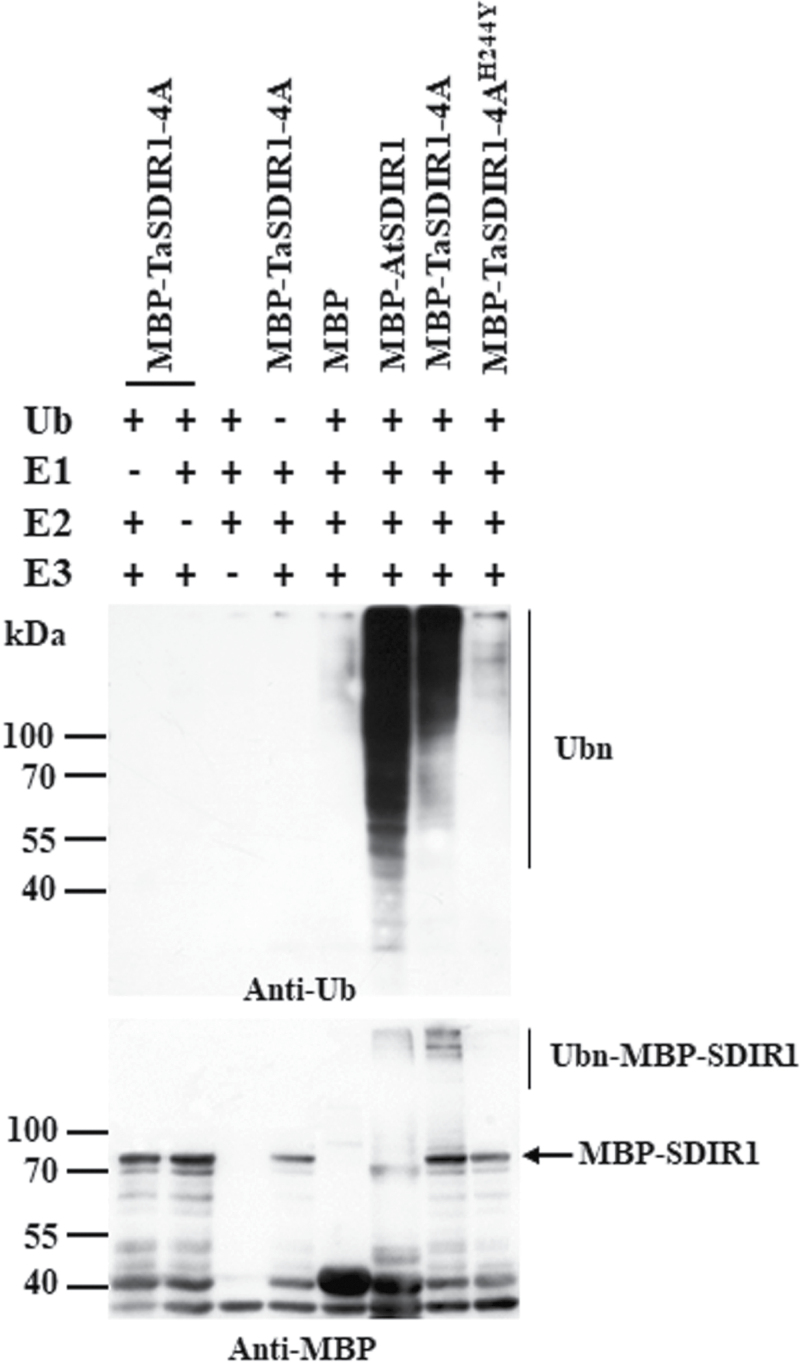
E3 ubiquitin ligase activity of wheat TaSDIR1-4A. The fusion proteins MBP-TaSDIR1-4A and its mutant form MBP-TaSDIR1-4A^H244Y^ were assayed for E3 activity in the presence of E1 (from wheat), E2 (UBCh5b), and 6×His tag ubiquitin (Ub). The molecular masses of the marker proteins are shown (kDa). MBP was used as a negative control and MBP-AtSDIR1 was used as a positive control. Samples were resolved by 10% SDS-PAGE. An anti-Ub antibody was used to detect His tag ubiquitin (top image), and the anti-MBP antibody was used for detection of maltose fusion proteins (bottom image).

Previous studies have shown that the RING finger-containing protein AtSDIR1 functions as an E3 ligase (Zhang *et al.*, 2007). An *in vitro* E3 ubiquitin ligase activity assay was therefore performed to test whether TaSDIR1-4A had E3 ligase activity, using AtSDIR1 as a positive control. Ubiquitination activity was observed in the presence of 6×His tag ubiquitin (Ub), E1 (from wheat), E2 (UBCh5b) and purified MBP-AtSDIR1 or MBP-TaSDIR1-4A proteins using an anti-Ub antibody (Fig. 1). An anti-MBP blot analysis also indicated that MBP-AtSDIR1 and MBP-TaSDIR1-4A were ubiquitinated. However, with MBP alone, or in the absence of E1, E2, E3, or Ub, no polyubiquitination was detected. The highly conserved RING finger domain of SDIR1, from Cys211 to Cys241 in Arabidopsis, has E3 ubiquitin ligase activity. The equivalent functional region in TaSDIR1-4A was from Cys221 to Cys251. The RING motif was essential for the E3 ligase activity of AtSDIR1. Our previous work had generated an allele with a single amino acid substitution by mutagenizing His-234 to Tyr (H234Y) in AtSDIR1, which abolished the E3 ligase activity (Zhang *et al.*, 2007). Presumably the mutation disrupted the RING domain. A similar mutation in TaSDIR1-4A, His-244-Tyr, was produced, and again an *in vitro* ubiquitination assay indicated that the E3 ligase activity was completely disrupted (Fig. 1). These results indicated that TaSDIR1-4A has E3 ligase activity and that an intact RING domain is required for enzyme activity.

### Sequence polymorphism assays, genetic mapping, and association analysis

**Fig. 2.**
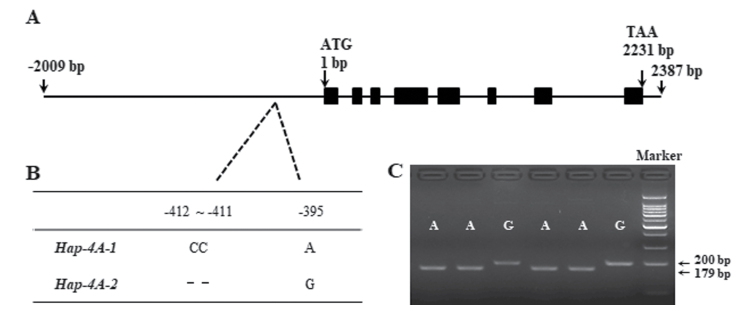
Nucleotide polymorphisms and development of a functional marker in wheat *TaSDIR1-4A*. (A) Schematic diagram of the *TaSDIR1-4A* structure. The ATG start codon was designated as position 1 bp. (B) Polymorphic sites were detected in the promoter regions of *TaSDIR1-4A*. (C) A dCAPS marker was developed based on the –395 bp single-nucleotide polymorphism (G/A). Digestion of the amplified 200-bp fragment with *Age* I produced fragments of 179 bp and 21 bp for accessions with the haplotype *Hap-4A-1* (A), whereas this fragment was not digested in accessions with the haplotype *Hap-4A-2* (G). Marker, 100-bp DNA ladder.

Sequence polymorphism assays indicated that there was no nucleotide polymorphism in the *TaSDIR1-4A* coding region, but two nucleotide variation sites were detected in the *TaSDIR1-4A* promoter region, a 2-bp InDel at –412 to –411 and a SNP at –395(Supplementary Fig. S2). Two haplotypes, *Hap-4A-1* and *Hap-4A-2*, were identified (Fig. 2A, B) and a functional marker (FM) based on SNP-395 (G/A) was able to distinguish between them (Fig. 2C). The marker contained a mismatch in the downstream primer that produced a recognition site for the restriction enzyme *Age* I.

Using diploid and tetraploid wheat relatives and a set of Chinese Spring nullisomic-tetrasomic lines, *TaSDIR1-4A* was found to be located on chromosome 4A (Supplementary Fig. S3A), and the functional marker was used to genotype the doubled-haploid (DH) lines in order to further map its position. Linkage analysis located a QTL for TGW between this functional marker and *AX-111611657* (Supplementary Fig. S3B). Using the same DH lines, a QTL for TGW and the TGW-related trait stem water-soluble carbohydrates had previously been identified in an adjacent interval flanked by the markers *Xgwm601* and *WMC420* (Yang *et al.*, 2007; Li *et al.*, 2012*a*). The marker *Xgwm601* was 3.56 cM from *TaSDIR1-4A*. Another QTL for the TGW-related trait chlorophyll content is also present in this region (Li *et al.*, 2010*a*). This indicated that *TaSDIR1-4A* might have a connection with grain weight.

**Fig. 3.**
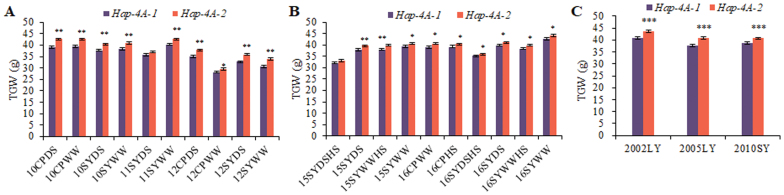
Comparisons of 1000-grain weight (TGW) in the two wheat *TaSDIR1-4A* haplotypes *Hap-4A-1* and *Hap-4A-2*. (A) Comparisons in Population 2 (262 accessions) grown in 10 different environments: either at Changping (CP) or Shunyi (SY), and under drought-stressed (DS) or well-watered (WW) conditions, planted in 2010, 2011, or 2012. (B) Comparisons in Population 1 (doubled-haploid) grown in 10 different environments: either at CP or SY, and under DS, WW, or heat-stressed (HS) conditions, planted in 2015 or 2016. (C) Comparisons in Population 4 (348 modern cultivars) grown in three different environments: SY planted in 2010 or Luoyang (LY) planted in 2002 or 2005. Data are means (±SE), *n*=3. Significant differences between the haplotypes were determined using Student’s *t*-test: *P<0.05, **P<0.01, and ***P<0.001.

Population 2 was used to examine the associations between the *TaSDIR1-4A* haplotypes and agronomic traits. Results of sequence polymorphism analysis of 262 accessions (Supplementary Table S4) indicated that variation at the *TaSDIR1-4A* locus was significantly associated with TGW in all the 10 environments that were examined (Table 1). *Hap-4A-1* and *Hap-4A-2* accounted for 36.0% and 64.0% of entries in this population, respectively. Accessions with *Hap-4A-2* had a higher mean TGW than accessions with *Hap-4A-1* (Fig. 3A), and thus it represented a more favorable haplotype. These differences were also found in Populations 1 and 4 (Fig. 3B, C).

**Table 1.** *TaSDIR1-4A* haplotypes associated with agronomic traits across 10 environments

Year planted	Site	Conditions	Effect on 1000-grain weight	
			*P*-value	PVE (%)
2010	CP	DS	3.56×10^–6^***	10.78
	CP	WW	8.38×10^–6^***	8.59
	SY	DS	4.09×10^–5^***	8.20
	SY	WW	1.74×10^–4^***	6.04
2011	SY	DS	0.0264*	4.41
	SY	WW	0.0013***	4.11
2012	CP	DS	4.57×10^–6^***	9.48
	CP	WW	0.03*	4.03
	SY	DS	7.48×10^–6^***	8.47
	SY	WW	3.03×10^–5^***	8.91

PVE, phenotypic variation explained. Sites: CP, Changping; SY, Shunyi. Conditions: DS, drought-stressed; WW, well-watered. Significant differences between the *TaSDIR1-4A* haplotypes were determined using Student’s *t*-test: *P<0.05, ***P<0.001.

### TaSDIR1-4A is a negative regulator of grain size

**Fig. 4.**
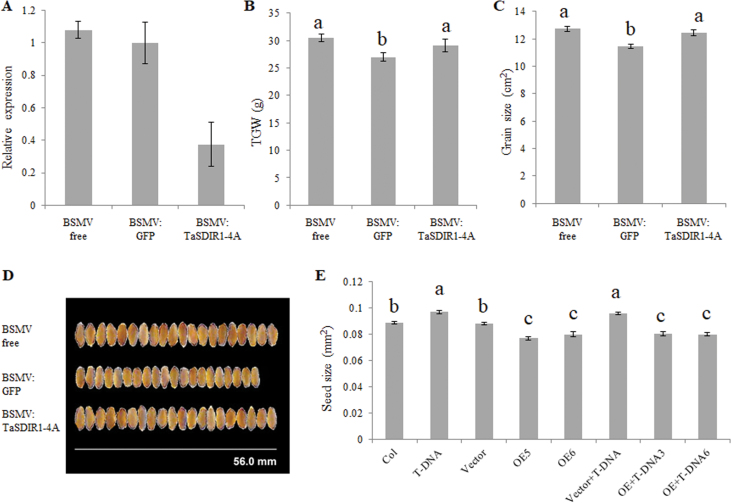
Phenotypic analysis of *TaSDIR1-4A*-silenced wheat and transgenic Arabidopsis. Virus-induced gene silencing (VIGS) of wheat was undertaken using a series of recombinant barley stripe mosaic virus (BSMV) vectors. (A) Relative expression of *TaSDIR1-4A* showing that transcription was lower in BSMV:TaSDIR1-4A plants than BSMV-free and BSMV:GFP plants. (B) Thousand-grain weight (TGW), (C) grain size, and (D) representative images of the grains. (E) Seed size in Arabidopsis plants in the Col-0 wild-type and T-DNA insertion mutant *sdir1-1* backgrounds overexpressing (OE) *TaSDIR1-4A*. Vector, empty vector control. Significant differences between means were determined using one-way ANOVA followed by Tukey’s test (*P*<0.05).

To further examine the role of *TaSDIR1-4A* in wheat we silenced its transcription by BSMV-mediated VIGS in the cultivar Chinese Spring. BSMV-associated chlorotic stripe mosaic symptoms appeared in newly emerged leaves 10 d post inoculation with the virus (Supplementary Fig. S4A). Flag leaves were harvested at anthesis and RT-PCR showed a 60% decline in transcripts in the BSMV:TaSDIR1-4A (silenced) lines compared with the BSMV:GFP lines (Fig. 4A). Quantitative real-time PCR was performed to confirm the specificity of knock-down of the expression of *TaSDIR1-4A*, no significant difference was observed in the expression of *TaSDIR1-4D* (the gene with the highest sequence similarity to *TaSDIR1-4A*) between the control and BSMV:TaSDIR1-4A lines (Supplementary Fig. S4B). Measurements after harvest showed that the silenced lines had larger grain size and higher TGW than the BSMV:GFP lines (Fig. 4B–D) whilst the BSMV-free lines had larger grain size and higher TGW than the BSMV-infected lines. Assuming that damage from BSMV infection would lead to smaller grain size and lower TGW, these results indicated that TaSDIR1-4A had a negative role in the determination of TGW.

To further confirm its effect on grain size and TGW, *TaSDIR1-4A* was overexpressed in the wild-type and the *sdir1-1* mutant of Arabidopsis and relative expression was determined by semi-quantitative PCR (Supplementary Fig. S5). The *sdir1-1* line had larger seeds whilst the *TaSDIR1-4A*-overexpression lines in both the wild-type and mutant background had smaller seeds than the non-transgenic controls (Fig. 4E).

### *TaSDIR1-4A* expression patterns

**Fig. 5.**
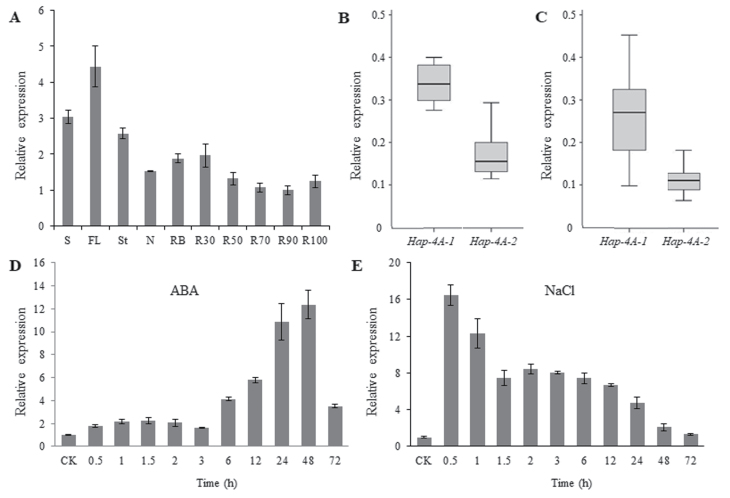
Expression patterns of the wheat *TaSDIR1-4A* haplotypes *Hap-4A-1* and *Hap-4A-2*. (A) Expression patterns of *TaSDIR1-4A* in different tissues of cv. H10 plants as measured by real-time PCR. S, spikes at flowering; FL, flag leaves; St, stems; N, nodes; RB, root bases; R30, roots from 0–30 cm depth; R50, roots from 30–50 cm; R70, roots from 50– cm; R90, roots from 70–90 cm; R100, roots from 90–100 cm. The values are relative to R90, which was set as 1. (B, C) Expression of *Hap-4A-1* and *Hap-4A-2* in (B) Population 1 and (C) Population 2. For each haplotype 12 accessions were randomly selected for measurement. (D) Expression patterns of *TaSDIR1-4A* following ABA treatment. Seedlings of cv. H10 at 2 weeks old were sprayed with 50 μM ABA solution and were sampled at 0 h (control, CK) and at 0.5–72 h. Expression is relative to the value in the control, which was set as 1. (E) Expression patterns of *TaSDIR1-4A* following NaCl treatment. Seedlings of cv. H10 at 2 weeks old were treated with 250 mM NaCl and were sampled at 0 h (CK) and at 0.5–72 h. Expression is relative to the value in the control, which was set as 1. In all cases *GAPDH* was used as the internal control. All data are means (±SE) of three biological replicates.

Because sequence polymorphism sites were located in the *TaSDIR1-4A* promoter region, we detected effects of variation on expression. We first performed real-time PCR on a range of tissues to determine expression patterns in wheat at the flowering stage, and found that *TaSDIR1-4A* was constitutively expressed in all tissues, with highest expression levels in the flag leaves (Fig. 5A). We then randomly selected 12 accessions of each of the two haplotypes from Populations 1 and 2 and planted them in the field (Supplementary Table S3). Flag leaves were collected at anthesis and the expression levels of *TaSDIR1-4A* were determined. The mean relative expression level of the *Hap-4A-1* accessions was 2.21-fold greater than that of the *Hap-4A-2* accessions in Population 1 (Fig. 5B), and 2.77-fold greater in Population 2 (Fig. 5C). In agreement with the results of the association analysis, accessions with *Hap-4A-1* had higher gene expression and lower TGW relative to those with *Hap-4A-2*, indicating that lower expression of *TaSDIR1-4A Hap-4A-2* resulted in higher TGW, and that TaSDIR1-4A plays a negative role in regulating TGW.

To determine whether TaSDIR1-4A plays a role in balancing the salt-stress response and yield, we examined responses to treatments with salt and ABA using real-time PCR. *TaSDIR1-4A* expression was induced by ABA (Fig. 5D) and transcription levels were at their highest (12-fold greater than the control) after 48 h of treatment. *TaSDIR1-4A* expression was also induced by salt and reached its highest level (16-fold greater than the control) after 0.5 h of treatment, following which it declined (Fig. 5E). These results demonstrated that *TaSDIR1-4A* was up-regulated in response to ABA and salt stress.

### TaERF3 regulates *TaSDIR1-4A* expression by binding to the promoter region

To further investigate the effects of the two polymorphic sites in the promoter region of *TaSDIR1-4A*, we first identified differences in *cis*-element between the two haplotypes. Using the Plant Transcription Factor Database (http://planttfdb.cbi.pku.edu.cn/), an ERF-binding site (CCCCGCCG) was identified at 395 bp upstream of the ATG translation start site in *Hap-4A-2*; the binding site was absent in *Hap-4A-1* due an A-to-G difference (CCCCACCG).

**Fig. 6.**
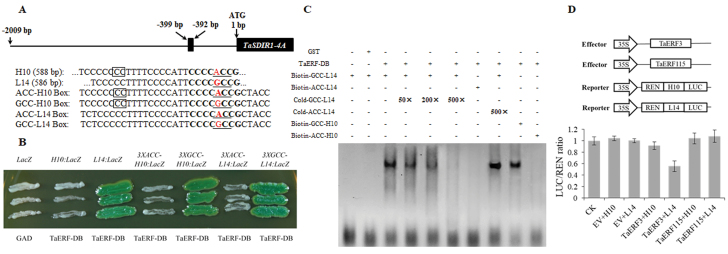
TaERF3 binds to the wheat *TaSDIR1-4A Hap-4A-2* promoter region. (A) Schematic diagram of the GCC Box (–399 to –392 bp) in the *Hap-4A-2* promoter. Letters in bold indicate the GCC Box regions, underlined letters indicate the GCC Box, letters in red are the single-nucleotide polymorphism sites (G/A), and the boxes indicate the 2-bp InDel sites. The fragments from the *TaSDIR1-4A* promoter regions of the cultivars H10 and L14 are shown, together with the 32-bp ACC Box from H10 (ACC-H10 Box), the same fragment but with A-to-G mutation (GCC-H10 Box), the 32-bp GCC Box from L14 (GCC-L14 Box), and the same fragment but with G-to-A mutation (ACC-L14 Box). (B) GAD-TaERF-BD activated expression in yeast of the *LacZ* reporter gene driven by the *Hap-4A-2* (L14) promoter and the GCC Box (either from H10 or L14), rather than the *Hap-4A-1* (H10) promoter and the 3×ACC Box (either from H10 or L14). (C) EMSA of the purified TaERF-BD protein and the GCC Box probe. Lane 1 contains biotin-labelled GCC-L14 Box probe, Lane 2 contains purified GST and biotin-labelled GCC-L14 Box probe, Lane 3 contains the TaERF-BD protein and biotin-labelled GCC-L14 Box probe, Lane 4 contains the TaERF-BD protein, biotin-labelled GCC-L14 Box probe, and 50× unlabeled (cold) GCC-L14 Box probe, Lane 5 contains the TaERF-BD protein, biotin-labelled GCC-L14 Box probe, and 200× cold GCC-L14 Box probe, Lane 6 contains the TaERF-BD protein, biotin-labelled GCC-L14 Box probe, and 500× cold GCC-L14 Box probe, Lane 7 contains the TaERF-BD protein, biotin-labelled ACC-L14 Box probe, Lane 8 contains the TaERF-BD protein, biotin-labelled GCC-L14 Box probe, and 500× unlabeled ACC-L14 Box probe, Lane 9 contains the TaERF-BD protein and biotin-labelled GCC-H10 Box probe, and Lane 10 contains the TaERF-BD protein and biotin-labelled ACC-H10 Box probe. The upper bands show that the TaERF-BD protein binds to the biotin-labelled GCC Box probe and the lower bands show the free probes. (D) Dual-luciferase assay of transformed tobacco leaves to examine the interaction between TaERFs and the *TaSDIR1-4A* promoter. *Agrobacterium* transformed with the vector pSoup harboring reporter and effector constructs, or with the empty vector (EV), were co-infiltrated into the leaves. Schematic diagrams of the effector and reporter constructs are shown. *TaERF3* or *TaERF115* was cloned into the effector construct pCAMBIA1300 and the promoter fragments from H10 or L14 were inserted into the reporter vector pGreen II 0800-LUC. Promoter activities are shown as the ratio of LUC to REN, and are relative to the control (CK) value, which was set to 1. Data are means (±SE) of three biological replicates.

Yeast one-hybrid assays were performed to determine whether TaERF would bind to the promoter region of *TaSDIR1-4A* by cloning the TaERF DNA binding region into the pB42AD vector. Each of the six fragments of the *TaSDIR1-4A* promoter region was cloned into pLacZi (Fig. 6A) and their interactions with TaERF-BD were tested by transforming it into yeast strain EGY48 cultured on selective media with x-α-gal and lacking Ura and Leu. In the presence of the *TaSDIR1-4A* promoter region from cv. L14 or the 3×GCC Box (either from cv. H10 or L14), TaERF-BD bound to and activated the *LacZ* reporter gene, whereas in the presence of the *TaSDIR1-4A* promoter region from H10 or the 3×ACC Box (neither from H10 or L14) it did not (Fig. 6A, B). These results suggested that the TaERF-BD could bind to the promoter of *Hap-4A-2* but not to that of *Hap-4A-1* due to the GCC/ACC Box difference rather than to the 2-bp InDel difference. These differences in binding might be responsible for the differences in the expression levels of two haplotypes.

Following induction and purification of the TaERF-BD protein, EMSAs were carried out to verify that it binds to the *TaSDIR1-4A* promoter region (Supplementary Fig. S6). Compared with the free probe, a slower-migrating DNA-binding band was detected with the addition of TaERF-BD and the biotin-labelled GCC Box probe (from either H10 or L14) (Fig. 6C), whereas there was no band in the GST control. The biotin-labelled DNA-binding band was diminished as the concentration of the unlabeled GCC Box probe was increased. The TaERF-BD could not bind to the biotin-labelled ACC Box probe from either H10 nor L14 and the unlabeled ACC Box probe did not compete with the labelled GCC Box probe. These results confirmed that the TaERF-BD could bind to the GCC Box region in the *Hap-4A-2* promoter but not to the ACC Box region in the *Hap-4A-1* promoter, and that this was due to the GCC/ACC Box difference rather than to the 2-bp InDel difference.

To examine which TaERF affects the expression of *TaSDIR1-4A*, we selected TaERF3, an ortholog of OsERF3, as the candidate protein based on previous reports that OsERF3 has EAR motif-mediated transcriptional repression activity and regulates yield-related genes (Ohta *et al.*, 2001; Kagale *et al.*, 2010; Wang *et al.*, 2011). TaERF3 is known to have relatively high expression levels in flower organs (Supplementary Fig. S7). It has been reported that OsERF115 can bind to the GCC box while not possessing transcriptional activation or repression activity, and *OsERF115* also shows higher expression levels during the grain development period and is associated with yield (Xu *et al.*, 2016). Hence TaERF115, an ortholog of OsERF115, was selected as the negative control. Dual luciferase assays showed that LUC activity was lower in the presence of both the TaERF3 effector and the *TaSDIR1-4A* promoter from L14 reporter constructs than in the negative control (Fig. 6D). In contrast, LUC activity was similar to the negative control in the presence of both the TaERF3 effector and the *TaSDIR1-4A* promoter from H10 reporter constructs, or in the presence of the TaERF115 effector and the *TaSDIR1-4A* promoter from H10/L14 reporter constructs. These results suggested that TaERF3 only acts as a transcriptional repressor of *TaSDIR1-4A* from L14 and not of *TaSDIR1-4A* from H10.

### Geographic and temporal distribution of *TaSDIR1-4A* haplotypes across different wheat production zones in China

**Fig. 7.**
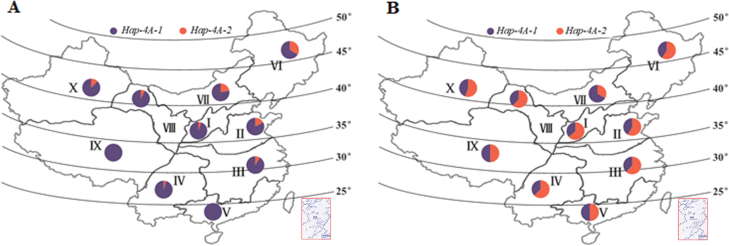
Distribution of the *TaSDIR1-4A* haplotypes *Hap-4A-1* and *Hap-4A-2* in 10 wheat-producing regions across China. (A) Proportions of the two haplotypes in 157 Chinese landraces and (B) in 348 modern cultivars. Winter wheat regions: I, Northern; II, Yellow and Huai River valleys; III, Low and middle Yangtze River valley; IV, Southwestern; V, Southern. Spring wheat regions: VI, Northeastern; VII, Northern; VIII, Northwestern. Spring–winter wheat regions: IX, Qinghai–Tibet; X, Xinjiang.

Genotypes of accessions in Population 3 (157 landraces) and Population 4 (348 modern cultivars) covering 10 different wheat production zones across the whole of China were used to investigate the geographic and temporal distributions of the *TaSDIR1* haplotypes. *Hap-4A-1* was the dominant haplotype in landraces from all 10 zones, especially Zones V and IX where all the accessions were of this haplotype (Fig. 7). In contrast, in modern cultivars the frequency of *Hap-4A-2* was generally higher, except in Zones V and IX, where the frequencies of the two haplotypes were approximately equal, and in Zone VII, the Northern Spring Wheat Zone, where the frequency of *Hap-4A-2* was lower than that of *Hap-4A-1*.

**Fig. 8.**
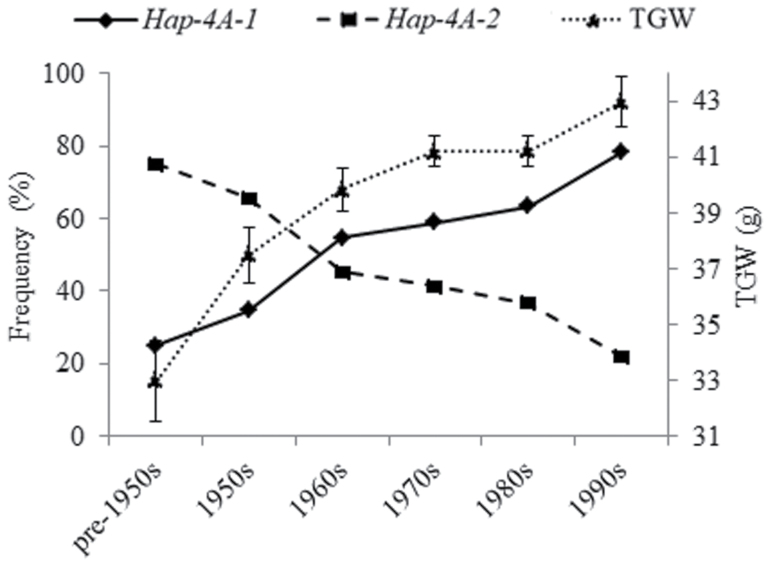
Frequencies of the wheat *TaSDIR1-4A* haplotypes *Hap-4A-1* and *Hap-4A-2* in Population 4 (348 modern cultivars) together with their corresponding 1000-grain weights plotted against the decades in which the cultivars were released. The data for TGW are from Hao *et al.* (2011), *n*=3.

The mean TGW in modern wheat varieties released in China from the pre-1950s to the 1990s has increased from 32.98 g to 42.97 g, and this has coincided with the proportion of haplotype *Hap-4A-2* increasing from 25.00% to 78.12% whilst that of *Hap-4A-1* declined from 75.00 to 21.88% (Fig. 8). This indicates that *Hap-4A-2* was positively selected in Chinese wheat breeding programs.

## Discussion

### TaSDIR1-4A is a functional RING finger E3 ligase with an unknown substrate

We have shown that TaSDIR1-4A is a functional RING finger E3 ligase (Fig. 1). Previous studies have demonstrated that AtSDIR1 targets SDIR1-INTERACTING PROTEIN1 for degradation in modulation of the response to salt stress and in ABA signaling (Zhang *et al.*, 2015). The rice RING-type protein GW2, which has E3 ubiquitin ligase activity, acts as a negative regulator of grain width, weight, and yield (Song *et al.*, 2007). TaGW2 also negatively regulates grain width and weight (Hong *et al.*, 2014; Jaiswal *et al.*, 2015; Simmonds *et al.*, 2016; Liu *et al.*, 2020). However, the substrate on which TaSDIR1-4A acts in its control of grain size, and any relationship between it and the TaGW2 substrate, requires further study.

### TaSDIR1-4A is a negative regulator of grain size

Previous studies have demonstrated that *SDIR1* is induced by stress and has a positive role in abiotic stress responses, but the role of SDIR1 in grain size development has been unknown (Zhang *et al.*, 2008; Liu *et al.*, 2013; Oh *et al.*, 2017). In this study, we have clearly shown that TaSDIR1-4A is a negative regulator of grain size and weight in two ways. Firstly, silencing of *TaSDIR1-4A* led to increased TGW in wheat and overexpression of *TaSDIR1-4A* in Arabidopsis resulted in reduced seed size (Fig. 4). Secondly, wheat accessions with the haplotype *Hap-4A-2* had lower *TaSDIR1-4A* expression and higher TGW than accessions with *Hap-4A-1* (Figs 3, 5B, C).

### TaSDIR1-4A might balance the relationship between stress responses and grain yield

Given that *TaSDIR1-4A* was up-regulated in response to ABA and salt-stress treatments (Fig. 5D, E) and that TaSDIR1-4A was a negative regulator of grain size (Figs 3, 4, 5B, C), we propose that TaSDIR1-4A may play a role in balancing responses to stress and yield. Under normal conditions, lower expression levels of *TaSDIR1-4A* may contribute to higher yield in wheat, whilst under abiotic stress conditions up-regulation of *TaSDIR1-4A* may help to overcome the stress but at the cost of reduced grain yield. This suggests that TaSDIR1-4A is the central control unit of complex networks that balance responses under constantly changing environmental conditions.

### TaERF3 affects the expression of *TaSDIR1-4A* haplotypes

Ethylene response factor (ERF) specifically binds to a GCC Box and has important functions in the control of grain size and yield (Ohto *et al.*, 2005; Aya *et al.*, 2014; Müller and Munné-Bosch, 2015). For example, OsERF3 can bind to the promoter of *LRK6*, a rice yield-related gene, which might lead to its differential expression (Kagale *et al.*, 2010; Wang *et al.*, 2011). Salt-responsive ERF1 (SERF1) is a negative regulator of grain size in rice (Schmidt *et al.*, 2014). And the closely related rice ERF genes *AP37* and *AP59* have different effects on yield (Oh *et al.*, 2009). Tobacco NtERF3 and Arabidopsis AtERF3, which has an ERF-associated amphiphilic repression (EAR) motif, are active repressors of transcription (Ohta *et al.*, 2001). Our results demonstrated that TaERF3, which also has EAR motif, could bind to the promoter region of the *TaSDIR1-4A* haplotype *Hap-4A-2* and repress its expression, but that it did not do so for *Hap-4A-1* (Fig. 6). Accessions possessing *Hap-4A-2* had lower *TaSDIR1-4A* expression levels and greater TGWs compared to those possessing *Hap-4A-1*. These findings suggest that TaERF3 is a positive regulator for TGW (Supplementary Fig. S8), but its biological functioning needs further study.

### A stable and effective molecular marker for breeding

Pyramiding elite alleles for important agronomic traits through marker-assisted selection is an effective approach to accelerate crop breeding. In this study, we designed primers specific to the A genome for detection of the two *TaSDIR1-4A* haplotypes. The dCAPS marker that we identified was based on a single-nucleotide polymorphism and it distinguished between the haplotypes associated with the differences in TGW. Hence, it can be used in future marker-assisted breeding for greater TGW, although we recognize that the frequency of the favorable *Hap-4A-2* allele in elite Chinese germplasms is already about 75%.

### Conclusions

The gene sequence of *TaSDIR1-4A* is conserved, and two nucleotide variations were identified in its promoter region, namely a 2-bp InDel and a SNP. Based on the SNP at position 395 (G/A), a functional marker was developed to distinguish the two haplotypes. Association analysis demonstrated that *Hap-4A-2* was the favorable haplotype for high 1000-grain weight (TGW), with genotypes possessing it having lower *TaSDIR1-4A* expression and higher TGW than those possessing *Hap-4A-1*. Phenotypic analysis of gene-silenced wheat and transgenic Arabidopsis demonstrated that TaSDIR1-4A is a negative regulator for grain size. The functional marker that we have developed for *Hap-4A-2* should be helpful for future molecular breeding in wheat.

## Supplementary data

Supplementary data are available at *JXB* online.

Fig. S1. Alignment of sequences of plant SDIR1 homologs and phylogenic analysis.

Fig. S2. Sequence alignment of *TaSDIR1-4A* between 32 wheat accessions.

Fig. S3. Chromosome location and genetic mapping of *TaSDIR1-4A*.

Fig. S4. Chlorotic stripe mosaic symptoms of BSMV-infected plants and the specificity of knock-down.

Fig. S5. Semi-quantitative PCR for *TaSDIR1-4A* expression levels in Arabidopsis.

Fig. S6. Expression and purification of the TaERF-BD protein.

Fig. S7. Expression patterns of *TaERF3* in different tissues of wheat at different stages of growth.

Fig. S8. A working model of the role of *TaSDIR1-4A* in determining 1000-grain weight.

Table S1. List of the 32 accessions of the wheat population used for the detection of nucleotide polymorphisms.

Table S2. Primers used in this study.

Table S3. Accessions possessing the different haplotypes of *TaSDIR1-4A* that were randomly selected from Populations 1 and 2.

Table S4. The 262 accessions of Population 2 and their *TaSDIR1-4A* haplotypes.

eraa271_suppl_Supplementary_Figure

eraa271_suppl_Supplementary_Table

## Data Availability

All data supporting the findings of this study are available within the paper and within its supplementary materials published online.
