# Supplementary material for: RING finger ubiquitin E3 ligase gene TaSDIR1-4A contributes to determination of grain size in common wheat
Source: J Exp Bot. 2020 Jun 1;71(18):5377–88. doi: 10.1093/jxb/eraa271 (PMC7501821; doi:10.1093/jxb/eraa271)
Supplement: eraa271_suppl_Supplementary_Figure [file eraa271_suppl_supplementary_figure.pdf]

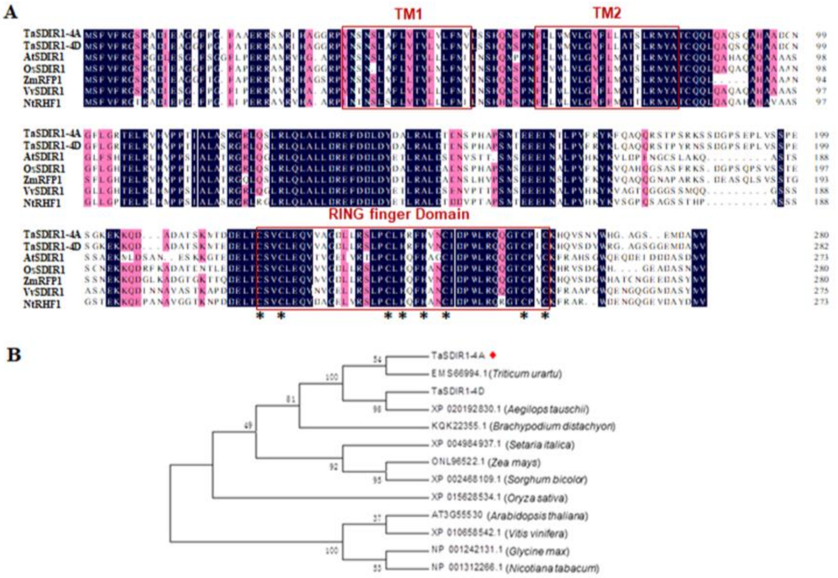

Fig. S1. Wheat TaSDIR1-4A belongs to the RING finger domain family.

(A) Alignment of amino acid sequences of plant SDIR1 homologs. The two putative transmembrane domains (TM1 and TM2) are marked in red frames. The highly conserved C3H2C3 RING finger domain is marked with a red frame, and asterisks indicate conserved Cys and His residues. (B) Phylogenetic tree of TaSDIR1-4A protein. The neighbor-joining tree was built with 1,000 bootstrap replicates in MEGA 5.05. TaSDIR1-4A is marked with red rhomboids. Ae, *Aegilops tauschii*; At, *Arabidopsis thaliana*; Bd, *Brachypodium distachyon*; Gm, *Glycine max*; Nt, *Nicotiana tabacum*; Os, *Oryza sativa*; Sb, *Sorghum bicolor*; Si, *Setaria italica*; Ta, *Triticum aestivum*; Tu, *Triticum urartu*; Vv, *Vitis vinifera*; Zm, *Zea mays*.

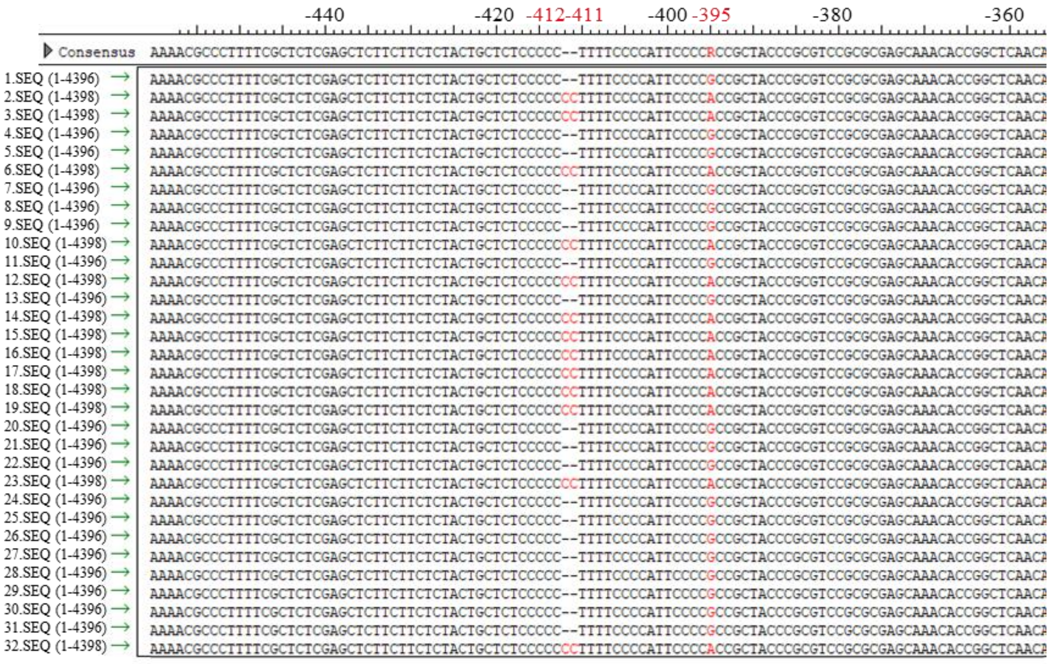

Fig. S2. Sequence alignment of *TaSDIR1-4A* between accessions in the diverse wheat population of 32 accessions.

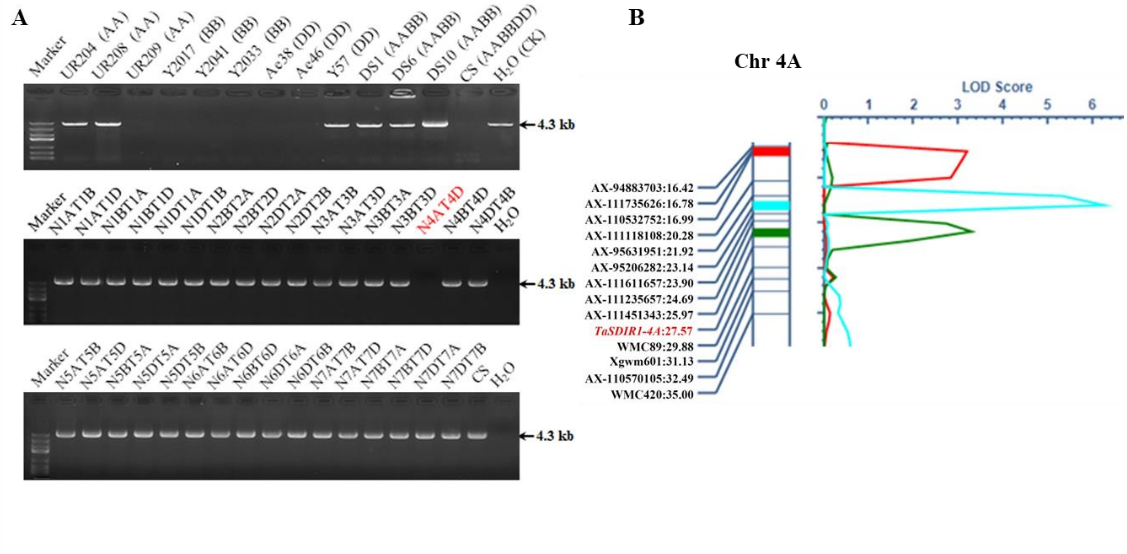

Fig. S3. Chromosome location and genetic mapping of *TaSDIR1-4A*.

(A) *TaSDIR1-4A* was located on chromosome 4A using wheat relatives with lower ploidy and a nullisomic-tetrasomic series of Chinese Spring. Marker, DNA Marker III. (B) Linkage of agronomic traits on chromosome 4A in Population 1 (DH lines). *TaSDIR1-4A* was flanked by *AX-111451343* and *WMC89*. Red line represents chlorophyll content. Cyan line represents TGW-related trait stem water soluble carbohydrates. Green line represents TGW.

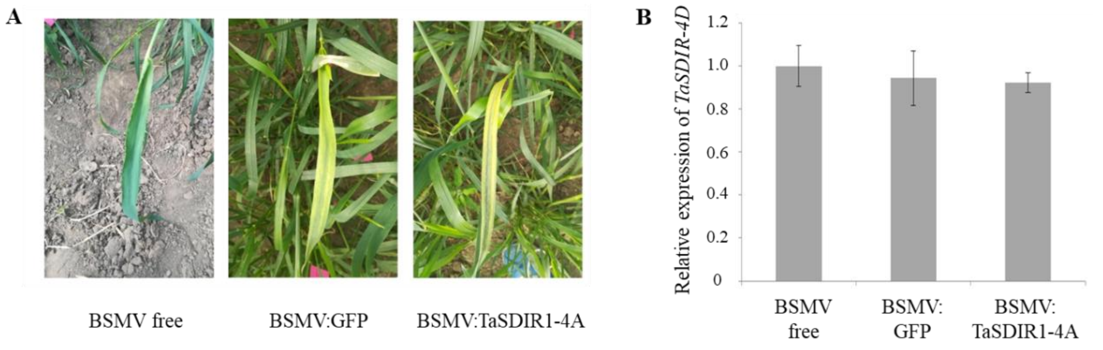

Fig. S4. Chlorotic stripe mosaic symptoms of BSMV-infected plants and specificity of knock down.

(A) BSMV-associated chlorotic stripe mosaic symptoms appeared on newly emerged leaves of BSMV:GFP and BSMV:TaSDIR1-4A incubated plants; BSMV-free plants showed no symptoms. (B) Expression pattern of *TaSDIR1-4D* in BSMV-free, BSMV:GFP and BSMV:TaSDIR1-4A incubated plants.

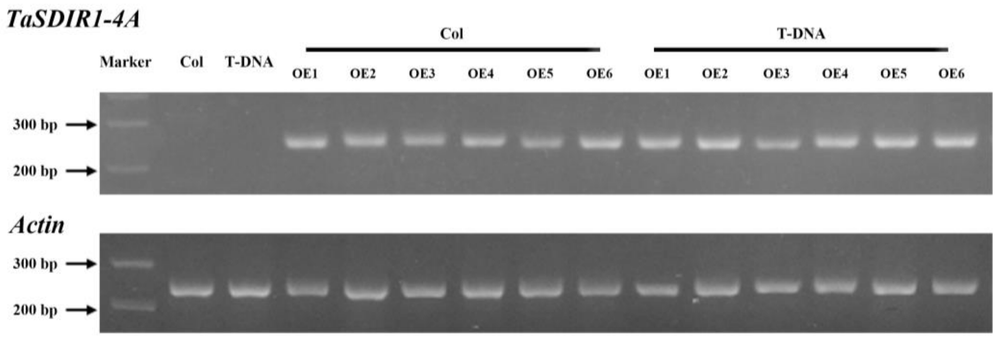

Fig. S5. *TaSDIR1-4A* expression levels in *Arabidopsis* detected by semi-quantitative PCR.

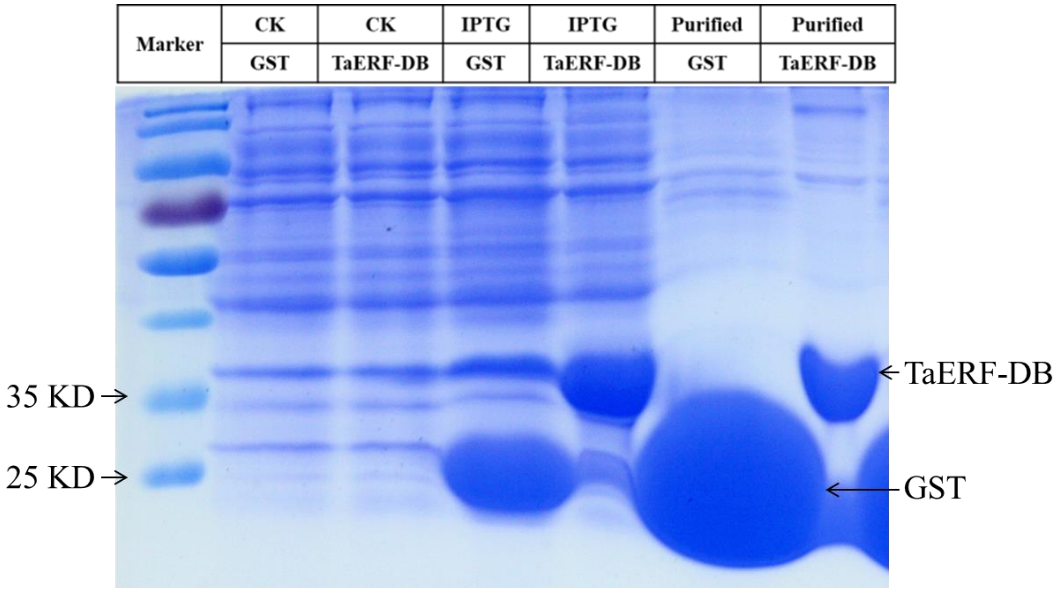

Fig. S6. Expression and purification of TaERF-DB protein.

Purified GST and TaERF-DB protein are marked by arrows. Lane 1 is a pre-stained protein marker. Lane 2 is the entire *E. coli* protein suspension that was transfected by an empty pGEX-4T1 vector before IPTG induction. Lane 3 is the entire *E. coli* protein suspension transfected by the pGEX-4T1 vector fused with TaERF-DB before IPTG induction. Lane 4 is the entire *E. coli* protein suspension transfected by an empty pGEX-4T1 vector after IPTG induction. Lane 5 is the entire *E. coli* protein suspension transfected by pGEX-4T1 vector fused with TaERF-DB after IPTG induction. Lane 6 is purified GST protein, and lane 7 is purified TaERF-DB protein.

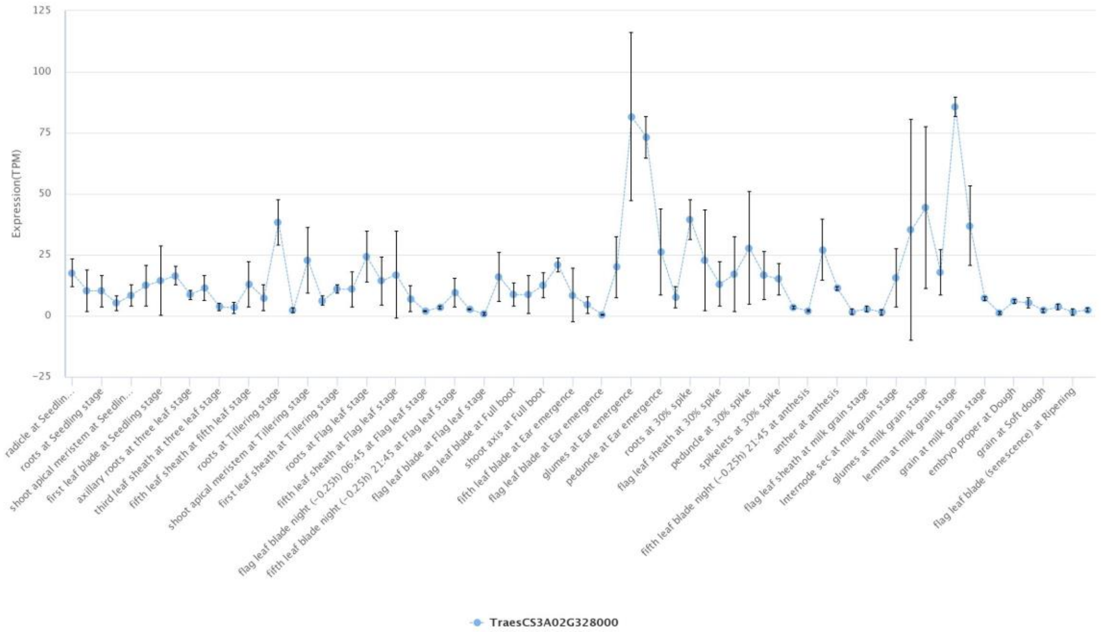

Fig. S7. Expression pattern of *TaERF3* (*TraesCS3A02G32800*) in wheat.
Data from website: https://wheat.pw.usda.gov/WheatExp/.

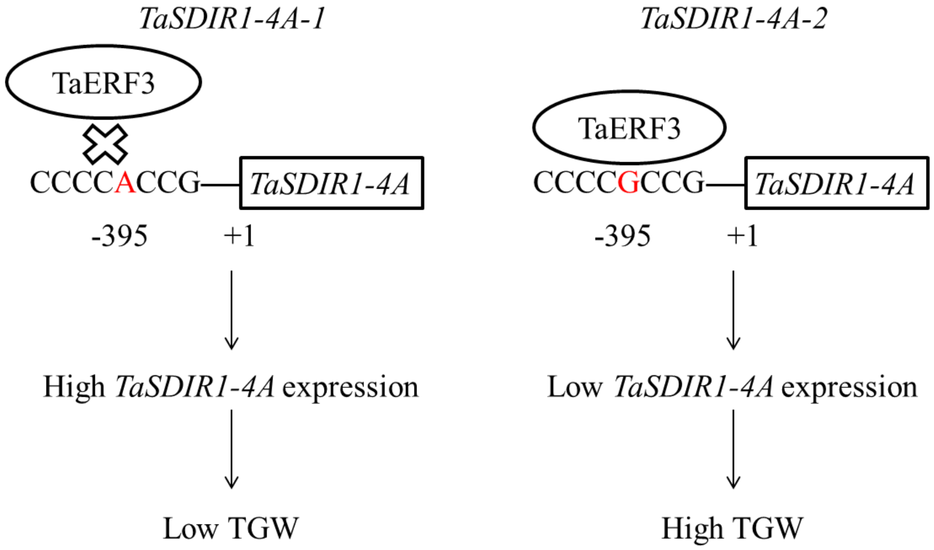

Fig. S8. A working model of *TaSDIR1-4A*.
