# Supplementary material for: RING finger ubiquitin E3 ligase gene TaSDIR1-4A contributes to determination of grain size in common wheat
Source: J Exp Bot. 2020 Jun 1;71(18):5377–88. doi: 10.1093/jxb/eraa271 (PMC7501821; doi:10.1093/jxb/eraa271)
Supplement: eraa271_suppl_Supplementary_Table [file eraa271_suppl_supplementary_table.pdf]

Table S1. Thirty-two accessions of the diverse wheat population used for nucleotide polymorphism detection

No.	Accession Name
1	PANDAS
2	An85 zhong124-1
3	Yanzhanyihao
4	Bawangbian
5	Beijing 10
6	Beijing 14
7	Canzhouxiaomai
8	Changwu 131
9	Chang 6878
10	Dali 1
11	Dan R8093
12	Fengkang 13
13	Jimai 41
14	Jimai 6
15	Jin 2148 - 7
16	Jinghe 8922
17	Linkang 5108
18	Baiqimai
19	Changle 5
20	Hongheshang
21	Beijing 8686
22	04-044
23	04-030
24	Chun22 9th-25
25	Ziganbaimangxian
26	Jingpin 10
27	Chun04 9th-5-1
28	Chun45 9th-50-1
29	Neixiang 188
30	Jing 411
31	Chinese Spring
32	Baicaomai

Table S2. Primers used for this study

Primer set	Nucleotide sequence (5' to 3')	Experimental purpose
AF1	GTTGGATCGTGAAGACGTACGACTACTTC	A genome-specific primers
AR1	CCTTCCTCTTGTGATGTTTATGCATCCAAAGG	A genome-specific primers
M13F	TGTAAAACGACGGCCAGT	Sequencing primers for A genome
M13R	CAGGAAACAGCTATGACC	Sequencing primers for A genome
AF2	CAAGAAGAAGGTTTCGGGATGAAGCAC	Sequencing primers for A genome

AF3	GCGACGTCGATTGTGTCAGAAATAAGAAGG	Sequencing primers for A genome
AF4	GGGATTCCAGAAATTGCAGTTTCGGTTG	Sequencing primers for A genome
AF5	CTTTTGTCGGATTGCTCAGAACTGTCTC	Sequencing primers for A genome
AR2	GTTGACCAATTTCCTATAAGCATCCTAGGAATCC	Sequencing primers for A genome
SDIR1-A-c-F	CTAGTCTAGAATGAGCTTTGTGTTCCGGGGCAGC	pCAMBIA1300 construction
SDIR1-A-c-R	ATGCGGTACCCACCATGGCATCCATTTCGCT	pCAMBIA1300 construction
SDIR-VIGS-F	TTCTTCCGTTGCTAGCATCACAAGAGGAAGGAACTGGGAACAC	VIGS vector construction
SDIR-VIGS-R	TTTTTTTTTAGCTAGCCATGTGAACTGCATTGATCCATGGTTGC	VIGS vector construction
TaERF-AD-DBF	TGCCTCTCCCGAATTCCTGCTAAAAGGAAGAGAAAGAACCAGTTCAG	pB42AD vector construction
TaERF-AD-DBR	CGAGTCGGCCGAATTCGGAGCCATAGGAGCTTCTTCTGG	pB42AD vector construction
LacZ-F	ATCTGTCGACCTCGAGGTGGAAGGGTGGCGTGTAAGAGAATATC	H10 & L14 pLacZi construction
LacZ-R	GAGCACATGCCTCGAGCCTCAACCGAAACTGCAATTTCTGGAATC	H10 & L14 pLacZi construction
SDIR1-A-qF	GCCAACCATAGCCCTTGCTTCGAGA	Real-time PCR
SDIR1-A-qR	CAGTCATAGAAGGAGCATGTGGGCTATTGTCA	Real-time PCR
pMAL-c5X-F	CGCGATATCGTCGACGGATCCATGAGCTTTGTGTTC	pMAL-c5X vector construction
pMAL-c5X-R	TTAATTACCTGCAGGGAATTCCACCATGGCATCCATTTC	pMAL-c5X vector construction
H-Y-F	CTTGCATCGGTTTTATGTGAACTGCATTGATCCATG	Mutant pMAL-c5X construction
H-Y-R	CATGGATCAATGCAGTTCACATAAAACCGATGCAAG	Mutant pMAL-c5X construction
AG-*Age* I-F	AGTTGCTCTAAGATCATAACTCGATACGGCCATCG	dCAPs Marker
AG-*Age* I-R	TTGCTCGCGCGGACGCGGGTA**C**CGG	dCAPs Marker
TaERF-4T1-DBF1	CGTGGATCCCCGGAATTCTCTGCTAAAAGGAAGAGAAAGAACCAGTTC	EMSA
TaERF-4T1-DBR1	GAGTCGACCCGGGAATTCAGGAGCCATAGGAGCTTCTTCTGG	EMSA
Hap-LUC-F	CGGTATCGATAAGCTTGTGGAAGGGTGGCGTGTAAGAGAATATC	Reporter Vector
Hap-LUC-R	TTGGCGTCTTCCATGGCCTCAACCGAAACTGCAATTTCTGGAATC	Reporter Vector
TaERF3-1300-F	GATCAAGCTTACTTGTACACCTGCTGCACCCATG	Effector Vector
TaERF3-1300-F	TCAGACTAGTGTCCTTTGTCTAGTCCTCGGAAGGC	Effector Vector
TaERF115-1300-F	GATCAAGCTTCATGAAGGTGAACACTAGGGCGATG	Effector Vector
TaERF115-1300-R	TCAGACTAGTCTGCCTAACATAGGGCAACAATGTCC	Effector Vector

Table S3. Accessions possessing different haplotypes of *TaSDIR1-4A* randomly selected from Population 1 & 2

**Haplotypes**	**Population 1**	**Population 2**
Hap-4A-1	DH1	DH23
	DH14	DH55

	DH66	DH57
	DH68	DH60
	DH69	DH79
	DH78	DH83
	DH88	DH106
	DH91	DH111
	DH119	DH121
	DH123	DH140
	DH132	DH147
	DH149	DH150
Hap-4A-2	Baicaomai	Chang 6154
	Baolin 9	Chang 6452
	Changzhi 620	Jingnong79-15
	Dali 1	Jinmai 53
	Dali 52	Luohan 6
	Fuzhuang 30	Nongda 20074
	Mazhamai	Shannongfu 63
	Mingxian 169	Yumai 48
	Shite 14	Yunhan 22-33
	Silenghonghulutou	Zhongda 91-9
	Xiaobaimai	Zhongmai 9
	Xifeng 16	Zhongzuo 60115
